# Mitotic Recombination: Why? When? How? Where?

**DOI:** 10.1371/journal.pgen.1000411

**Published:** 2009-03-13

**Authors:** Matthew C. LaFave, Jeff Sekelsky

**Affiliations:** 1 Curriculum in Genetics and Molecular Biology, University of North Carolina, Chapel Hill, North Carolina, United States of America; 2 Cell and Molecular Biology Training Program, University of North Carolina, Chapel Hill, North Carolina, United States of America; 3 Department of Biology, University of North Carolina, Chapel Hill, North Carolina, United States of America; The University of North Carolina at Chapel Hill, United States of America

DNA damage repair, loss of heterozygosity, and chromosome rearrangement are important aspects of genome stability, and all are tied to mitotic recombination. Despite the importance of mitotic recombination, the most basic questions about this process remain poorly understood. This is in part because mitotic recombination, in contrast to meiotic recombination, is rare on a per cell division basis [1]. A number of systems have been devised to detect or select for mitotic recombination. In this issue of *PLoS Genetics*, Lee et al. [2] describe a novel system that represents a major step forward in the study of spontaneous mitotic recombination events. Their studies have given us new insights into the why, when, how, and where of mitotic recombination.

Mitotic recombination was first described by Stern in his classic *Drosophila* experiments [3]. For Stern, “recombination” referred only to reciprocal crossovers (RCOs) (Figure 1A). A severe limitation of most RCO assays is that only one of the two reciprocal products can be recovered. Barbera and Petes [4] devised a clever method to recover both products of RCOs in *Saccharomyces cerevisiae*. They used this method to measure rates of spontaneous and induced mitotic recombination. Lee et al. have brought increased power to this assay by performing it in diploids with ∼0.5% heterology between the sequences of homologous chromosomes. This design allowed mapping of RCOs at high resolution, and also allowed study of another aspect of recombination—gene conversion (Figure 1C and 1D). Their analysis led to several key findings that provide unique and sometimes surprising insights into questions about mitotic recombination.

**Figure 1:**
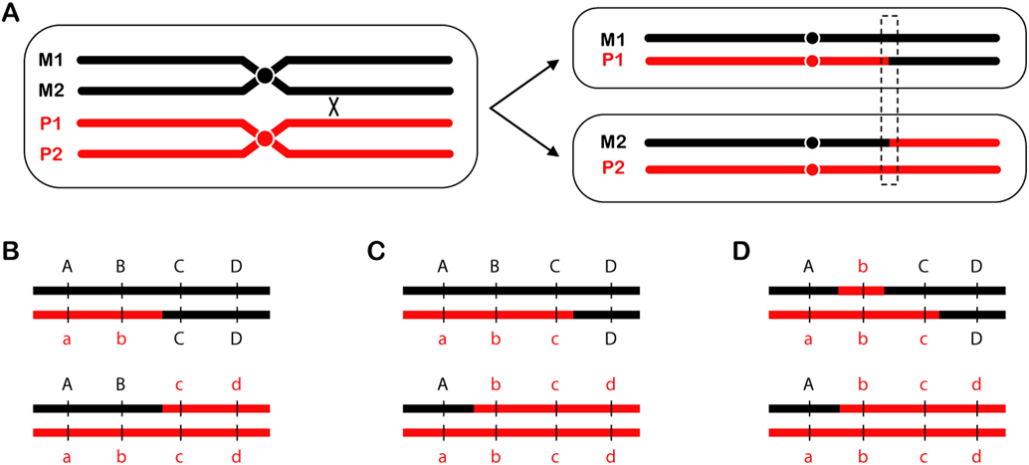
Reciprocal crossovers and gene conversion. (A) An RCO is depicted between chromatids of two homologous chromosomes. One segregation pattern results in daughter cells that have become homozygous for the sequence distal to the crossover site. (B–D) A close-up view of the region outlined by the dotted box, showing different gene conversion tract configurations detectable using markers *a* through *d*. (B) No conversion tract, either because there was no gene conversion or the tract was too small to be detected with the markers available. All markers are still present in a 2∶2 ratio. (C) A typical gene conversion event produces a tract that alters some of the markers (*b* and *c*) to a 3∶1 ratio. Note that conversion tracts can only be detected if both reciprocal products (i.e., both daughter cells) are recovered and analyzed, as done by Lee et al. (D) Lee et al. observed some tracts that were wholly or partially 4∶0. In the example shown here, marker *b* has segregated 4∶0, but marker *c* has segregated 3∶1; this is therefore a 4∶0/3∶1 hybrid gene conversion tract.

## Why?

Why does mitotic recombination, which can be detrimental, occur? Answering this question begins with asking what initiates the process. Lee et al. suggest that most spontaneous RCOs are initiated by DNA double-strand breaks (DSBs). Recombinational repair of a DSB requires a template; when the homologous chromosome serves that role, it provides the opportunity for an RCO. Since there is also evidence that single-stranded nicks and gaps are recombinogenic [5], it is likely that several types of DNA lesions may be important for spontaneous mitotic recombination events. In addition, some recombinogenic agents (such as ultraviolet radiation) are thought to produce nicks that result in DSBs when the nicked DNA is replicated [6]. Thus, the question of why becomes tied up with the question of when.

## When?

At what point in the cell cycle does mitotic recombination occur? Whereas meiotic recombination occurs during meiosis, most mitotic recombination probably does not occur during mitosis, but during interphase. Analysis of gene conversion tracts associated with RCOs provides clues about when during interphase mitotic recombination takes place. Gene conversion is a nonreciprocal exchange of genetic information. Normal gene conversion between homologous chromosomes produces a 3∶1 ratio of alleles (Figure 1C); however, Lee et al. also detected 4∶0 and 3∶1/4∶0 hybrid tracts (Figure 1D). Lee et al. argue that a 4∶0 tract most likely results when a break occurs prior to DNA replication, but repair takes place after replication. As depicted in Figure 8 of Lee et al., replication of a broken chromatid results in sister chromatids that are both broken at the same position. Since both are broken, the homologous chromosome must be used as a repair template. If both broken chromatids repair off the homologous chromosome, a 4∶0 or 4∶0/3∶1 hybrid is produced, depending on whether or not both tracts are identical. The high frequency of 4∶0 and 3∶1/4∶0 hybrid tracts suggests that a considerable fraction of the breaks that results in RCOs occur before replication.

## How?

What is the molecular mechanism by which mitotic recombination is accomplished? The results presented by Lee et al. suggest that RCOs with different gene conversion tract lengths may be produced by different mechanisms. Short tracts may result from a DSB repair pathway involving heteroduplex formation followed by mismatch repair [1]. A heteroduplex is a region of DNA composed of strands that are derived from two different chromosomes. Polymorphisms between the two chromosomes will result in mismatches, and repair of these mismatches can result in gene conversion. Although this mechanism has been proven to be important for meiotic gene conversion, in which the conversion tracts are usually 1–2 kb long, evidence that it can produce the very long conversion tracts (average of 12 kb) observed by Lee et al. is lacking.

Lee et al. argue that the very large conversion tracts (some up to 100 kb in length) may reflect a different process, gap repair [7]. If a DSB is processed to form a gap, or if two DSBs occur on the same chromatid, the potential for extensive heteroduplex formation is eliminated. Instead, the entire gap is filled using the homologous chromosome as a template, resulting in a long patch of gene conversion. If these long tracts are indeed produced by gap repair, it raises the question of whether the RCOs associated with these tracts arise from a double-Holliday junction (DHJ) intermediate. Meiotic crossovers in *S. cerevisiae* involve a DHJ intermediate [8], but it is unknown whether this structure can be produced across a long gap.

## Where?

Are there hotspots for mitotic recombination as there are for meiotic recombination? It is believed that some sites are hotter for DSB formation than others. Common fragile sites (CFSs), regions of the genome prone to chromosomal DSBs, are a normal feature of mammalian chromosomes, and analogous regions have been identified in yeast [9]. Most studies of CFSs have relied on the use of replication inhibitors to increase the frequency of breaks, followed by cytological detection [10]. In contrast, the approach taken by Lee et al. allows high-resolution molecular mapping of hotspots for mitotic recombination. They found that sites of RCOs, and therefore the initial sites of spontaneous damage, were nonrandomly distributed. Furthermore, the authors uncovered evidence for the existence of one region with elevated RCOs. This is exceptionally interesting because it represents a region prone to spontaneous rather than induced DSBs. The fact that such a hotspot could be detected by examining only 1% of the genome makes this discovery more intriguing still. It will be exciting to see if other such hotspots for spontaneous damage and mitotic recombination exist, and what role they play in genome stability. Taking advantage of the flexibility of the assay used by Lee et al., such as focusing on other regions of the genome or incorporating DNA repair mutants, will surely aid in future studies.

Many questions concerning mitotic recombination remain to be answered. Perhaps the most basic of these is what makes certain regions more prone to mitotic breakage and recombination than others? The approach of Lee et al. can address this question—and others—in an exciting new way by focusing on regions prone to spontaneous damage, as opposed to induced damage. These and other studies of mitotic recombination, a process both fundamental and far reaching, promise to continue to provide interesting insights into causes of genome instability.
